# The night matters: sleep quality and evening chronotype associated with clinical severity of psoriasis

**DOI:** 10.3389/fendo.2026.1788526

**Published:** 2026-03-11

**Authors:** Ludovica Verde, Martina Galasso, Giuseppe Annunziata, Matteo Megna, Lucia Gallo, Luca Potestio, Annamaria Colao, Giovanna Muscogiuri, Luigi Barrea

**Affiliations:** 1 Centro Italiano per la cura e il Benessere del Paziente con Obesità (C.I.B.O), Unità di Endocrinologia, Diabetologia e Andrologia, Dipartimento di Medicina Clinica e Chirurgia, Università degli Studi di Napoli Federico II, Naples, Italy; 2 Division of Endocrinology, Department of Medicine, The University of Arizona College of Medicine, Tucson, AZ, United States; 3 Department for the Promotion of Human Sciences and Quality of Life, San Raffaele Roma Open University, Rome, Italy; 4 Section of Dermatology, Department of Clinical Medicine and Surgery, University of Naples Federico II, Naples, Italy; 5 Dipartimento di Medicina Clinica e Chirurgia, Unità di Endocrinologia, Diabetologia ed Andrologia, Università degli Studi di Napoli Federico II, Naples, Italy; 6 Cattedra Unesco “Educazione Alla Salute E Allo Sviluppo Sostenibile”, Università degli Studi di Napoli Federico II, Naples, Italy; 7 Dipartimento di Psicologia e Scienze della Salute, Università Telematica Pegaso, Naples, Italy

**Keywords:** adiposity, chronotype, circadian rhythms, inflammation, obesity, psoriasis, sleep

## Abstract

**Introduction:**

Psoriasis is a chronic inflammatory skin disease associated with metabolic dysfunction and obesity. Poor sleep quality appears more prevalent in psoriasis, yet the role of chronotype remains largely unexplored. We evaluated whether patients with psoriasis more frequently exhibit poor sleep quality and evening chronotype compared with healthy controls and assessed the impact of these factors on psoriasis clinical severity.

**Methods:**

In this cross-sectional study, 213 adults with psoriasis and 213 age- and sex-matched healthy controls were included. Anthropometric measures (body mass index [BMI], waist circumference [WC]), metabolic indices (fatty liver index [FLI], visceral adiposity index [VAI]), inflammation (C-reactive protein [CRP]), sleep quality (Pittsburgh Sleep Quality Index [PSQI]), and chronotype (Morningness–Eveningness Questionnaire [MEQ]) score were assessed. Psoriasis clinical severity was evaluated using the Psoriasis Area Severity Index (PASI).

**Results:**

Compared with controls, patients with psoriasis showed poorer sleep quality (PSQI 9.3 ± 4.9 vs 6.8 ± 4.7 score; p < 0.001) and lower MEQ scores (39.2 ± 19.8 vs 43.1 ± 19.2 score; p = 0.032), indicating a tendency toward the evening chronotype. Within the psoriasis cohort, poor sleepers (PSQI score > 5) had significantly higher PASI than good sleepers (PSQI score ≤ 5; 10.3 ± 6.51 vs 2.3 ± 1.6 score; p < 0.001). Evening chronotype patients exhibited the highest PASI score (11.1 ± 6.3 score), exceeding both morning (1.7 ± 1.1 score) and intermediate chronotypes (3.9 ± 1.2 score) (all p < 0.001). PASI score correlated positively with BMI (r = 0.383), WC (r = 0.478), PSQI score (r = 0.766), CRP levels (r = 0.435), FLI score (r = 0.457), and VAI score (r = 0.627), and inversely with MEQ score (r = −0.781) (all p < 0.001). Stepwise multiple regression identified MEQ score, PSQI score, FLI score, VAI score, CRP levels, WC, and smoking status as independent predictors of PASI score, explaining 81% of its variance (adjusted R² = 0.806). MEQ score showed the strongest independent inverse association with PASI score (p < 0.001). PSQI score (p < 0.001), VAI score (p < 0.001), CRP levels (p < 0.001), WC (p = 0.049), and smoking status (p = 0.009) were positively associated with PASI score, while FLI score showed a modest inverse association (p = 0.019). ROC analysis showed a PSQI score > 10 predicted a moderate-to-severe PASI score (sensitivity 75.7%, specificity 83.9%, AUC 0.856, p < 0.001), while a MEQ score ≤ 25 showed higher accuracy (sensitivity 98.6%, specificity 90.9%, AUC 0.979, p < 0.001).

**Conclusion:**

Patients with psoriasis more frequently display poor sleep quality and evening chronotype compared to healthy controls. Both factors are independently associated with greater disease severity, regardless of adiposity, supporting the value of routine assessment of sleep quality and chronotype to guide personalized management strategies.

## 1 Introduction

Psoriasis is a chronic immune-mediated inflammatory skin disease affecting approximately 2–3% of the adult population worldwide (1). Beyond its cutaneous manifestations, psoriasis is increasingly recognized as a systemic condition associated with metabolic comorbidities, including obesity, insulin resistance, dyslipidemia, metabolic-associated fatty liver disease, and cardiovascular risk (1). Excess adiposity, particularly visceral fat accumulation, plays a key role in sustaining low-grade systemic inflammation through the release of pro-inflammatory cytokines and adipokines, thereby contributing to both the development and severity of psoriasis (2).

Sleep disturbances are common in patients with chronic inflammatory diseases, and growing evidence suggests that individuals with psoriasis experience poorer sleep quality compared with the general population (3–5). Pruritus, pain, psychological distress, and systemic inflammation may disrupt sleep architecture, leading to impaired sleep duration and efficiency (6). In turn, poor sleep quality has been linked to metabolic dysregulation, increased inflammatory burden, and exacerbation of immune-mediated diseases, suggesting a bidirectional relationship between sleep and psoriasis activity (6). However, sleep quality has often been considered in isolation, without accounting for broader circadian factors that may influence disease severity (3–5).

Chronotype, defined as an individual’s circadian preference for morning or evening activity, reflects endogenous circadian rhythms and behavioral patterns related to sleep–wake timing (7). The evening chronotype has been associated with adverse metabolic profiles, including obesity, insulin resistance, visceral adiposity, and systemic inflammation. Moreover, circadian misalignment and evening preference have been implicated in immune dysregulation and heightened inflammatory responses (7), which are central to psoriasis pathophysiology (1). Despite these mechanistic links, the role of chronotype in psoriasis has received limited attention, and its potential contribution to disease severity remains largely unexplored (8–10).

Adiposity, sleep quality, and circadian preference are closely interconnected and may synergistically influence inflammatory pathways (11). Individuals with overweight or obesity are more likely to exhibit poor sleep quality and evening chronotype, which may further aggravate metabolic dysfunction and inflammatory load (12). In the context of psoriasis, this interplay may help explain the heterogeneity in disease severity observed among patients with similar levels of adiposity. However, few studies have simultaneously examined these factors within a single analytical framework, particularly in populations at high metabolic risk.

Therefore, the present study aimed to evaluate sleep quality and chronotype in adults with psoriasis compared with healthy matched controls and to investigate their associations with psoriasis clinical severity.

## 2 Methods

### 2.1 Study design and population

This cross-sectional case-control observational study was conducted at the Outpatient Clinic of the Section of Dermatology, University of Naples Federico II (Italy), in accordance with the Declaration of Helsinki. All participants provided written informed consent prior to enrollment.

Patients older than 18 years and with a diagnosis of mild-to-severe psoriasis lasting for at least 6 months were enrolled, while patients with pustular, erythrodermic, or psoriatic arthritis or receiving any systemic treatment for psoriasis, including acitretin, ciclosporin, methotrexate, dimethyl fumarate, phototherapy, biologics, or small molecules, for at least 3 months were excluded. Exclusion criteria also included alcohol abuse. Furthermore, they had not received any drug therapy known to affect carbohydrate or lipid metabolism for the 6 months before. All patients were checked and recorded.

Non-psoriatic subjects were recruited as healthy controls among hospital volunteers and employees from the same geographical area. Controls were matched based on age and sex. The exclusion criteria for controls were the same as for the patients, with the additional criterion that controls had no previous diagnosis of psoriasis.

**Figure 1:**
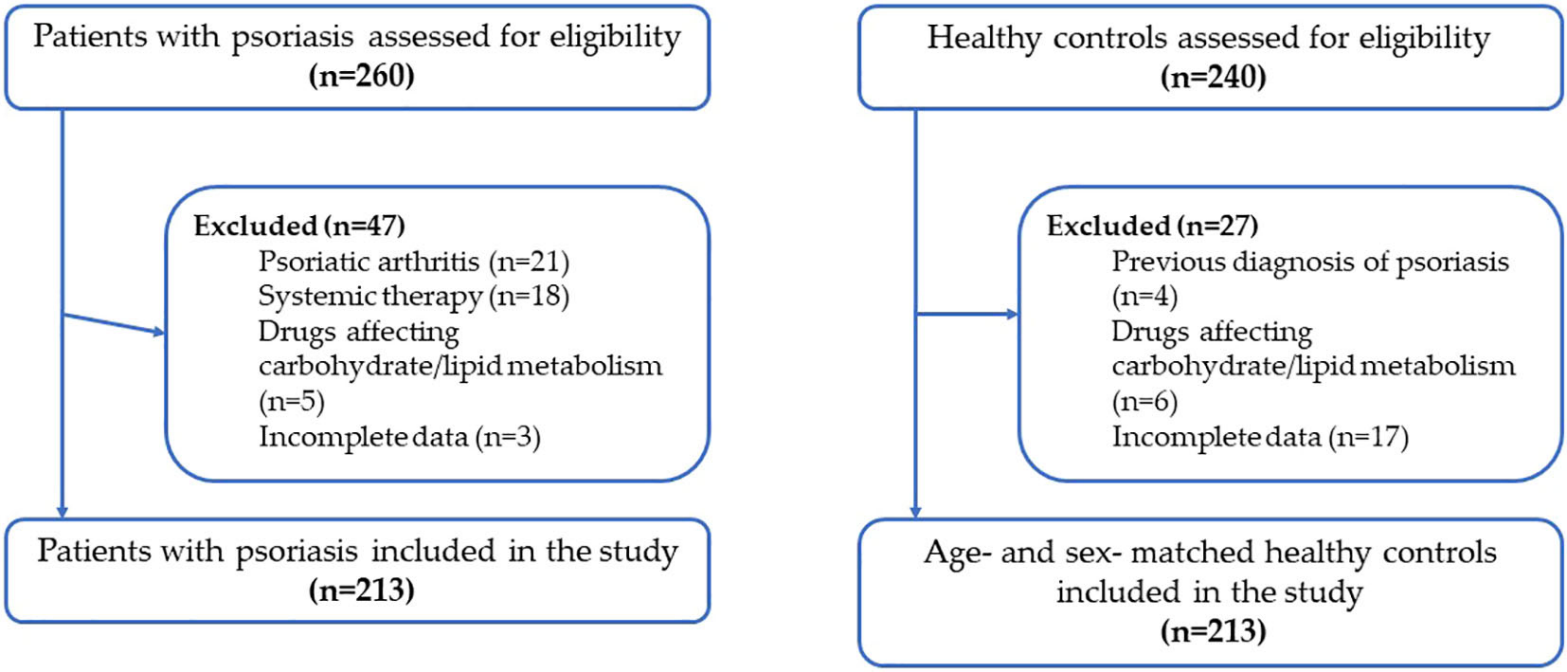
Flow diagram of participant selection.

A flow diagram summarizing the screening, exclusion, and final inclusion of participants in both the psoriasis and control groups is shown in **Figure 1**. Only participants with complete data for all clinical, metabolic, sleep, and chronotype variables were included in the analysis.

### 2.2 Psoriasis assessment

Psoriasis clinical severity was evaluated using the Psoriasis Area and Severity Index (PASI), a well-established tool for quantifying disease clinical severity (13). The PASI assesses four body regions—head and neck, upper limbs, trunk, and lower limbs—based on the degree of erythema, scaling, and plaque thickness. Scores can range from 0 to 72, with higher scores reflecting more severe disease. Psoriasis clinical severity was categorized according to PASI score as mild (PASI score ≤ 10), moderate (PASI score 11–19), or severe (PASI score ≥ 20), in line with previously published criteria (14).

### 2.3 Lifestyle habits

Physical activity and smoking status were assessed using standardized questionnaires, as previously reported (15). Participants were considered physically active if they engaged in at least 30 minutes of aerobic exercise per day (yes/no). Smoking status was categorized as current smokers (≥1 cigarette/day), former smokers (quit ≥1 year before the interview), or non-smokers; former and non-smokers were grouped together for analysis.

### 2.4 Anthropometric assessment

Anthropometric measurements were conducted between 8:00 and 10:00 a.m. following an overnight fast, with participants wearing light clothing and no shoes. Body weight was recorded to the nearest 0.1 kg using a calibrated beam scale, and height was measured to the nearest 0.5 cm with a wall-mounted stadiometer. Body mass index (BMI) was calculated as weight in kilograms divided by height in meters squared (kg/m²). Waist circumference (WC) was measured to the nearest 0.1 cm at the natural waist; if the waist crease was not visible, the midpoint between the lower rib margin and iliac crest was used.

### 2.5 Sleep assessment

Sleep quality was assessed using the PSQI questionnaire (16). Participants were categorized as poor sleepers if their PSQI score was >5 and as good sleepers if the score was ≤5 (16).

### 2.6 Chronotype assessment

Chronotype was evaluated using the Horne–Östberg Morningness–Eveningness Questionnaire (MEQ), which includes 19 items assessing preferred sleep–wake timing and peak periods of daily performance (17). MEQ scores range from 16 to 86, with participants classified as morning (59–86), intermediate (42–58), or evening chronotype (16–41) based on their total score (17).

### 2.7 Cardiometabolic indices

The Visceral Adiposity Index (VAI) and Fatty Liver Index (FLI) were used as non-invasive markers of metabolic dysfunction, visceral fat accumulation, and systemic inflammation, which may contribute to psoriasis clinical severity (18, 19).

The VAI was calculated using sex-specific formulas, with triglyceride (TG) and high-density lipoprotein (HDL) levels expressed in mmol/L.


Males: VAI = [WC/39.68+(1.88×BMI)]×(TG/1.03)×(1.31/HDL)

Females: VAI = [WC/36.58+(1.89×BMI)]×(TG/0.81)×(1.52/HDL)

The FLI was computed as


FLI = eL/(1 + eL) × 100, L = 0.953 × loge TG + 0.139 BMI + 0.718 × loge γGT + 0.053 × WC −15.745

### 2.8 Assay methods

Following an overnight fast of at least 8 hours, blood samples were collected between 8:00 and 10:00 a.m. and stored at −80 °C until analysis. All biochemical measurements were performed using a Roche Modular Analytics System in the Central Biochemistry Laboratory of our institution. Low-density lipoprotein (LDL) and HDL cholesterol levels were measured using a direct homogeneous enzymatic assay for quantitative determination. C-reactive protein (CRP) levels were determined with a nephelometric assay with CardioPhase high-sensitive from Siemens Healthcare Diagnostics (Marburg, Germany). The intra-assay coefficients of variation (CV) for CRP were <3%; the low detection limit was >0.1 mg/L.

### 2.9 Statistical analysis

Only participants with complete data for all clinical, metabolic, sleep, and chronotype variables were included in the analysis. Data are presented as mean ± standard deviation for continuous variables and as counts and percentages for categorical variables. Comparisons between patients with psoriasis and matched controls were performed using Student’s t-test for normally distributed continuous variables and the chi-square test for categorical variables. For paired categorical variables, the McNemar test was applied. Differences across sleep quality groups (PSQI score ≤ 5 *vs* > 5) and chronotype categories (morning, intermediate, evening) were evaluated using one-way ANOVA with Bonferroni *post-hoc* correction or chi-square tests, as appropriate. Pearson correlation coefficients were calculated to examine associations between PASI score and clinical, metabolic, and sleep-related parameters. Stepwise multiple linear regression analysis was conducted with PASI score as the dependent variable, including age, BMI, WC, MEQ score, PSQI score, CRP levels, FLI score, and VAI score as candidate predictors. Assumptions of linear regression, including normality of residuals, linearity, homoscedasticity, and independence of errors, were verified. Multicollinearity was assessed using variance inflation factor (VIF) and tolerance statistics. Variables retained at each step of the model were reported, and those included in the final model were considered independent predictors of psoriasis clinical severity. Model performance was evaluated using adjusted R². Receiver operating characteristic (ROC) curve analysis was performed to assess the predictive accuracy of PSQI and MEQ scores for moderate-to-severe psoriasis (PASI score ≥ 10). Statistical analysis were conducted using SPSS version 22 (IBM Corp., Armonk, NY), with a significance threshold set at p < 0.05.

## 3 Results

Characteristics of patients with psoriasis and matched controls are shown in **Table 1**.

**Table 1:** Characteristics of patients with psoriasis and matched healthy controls.

Parameter	Psoriasis (n = 213)	Controls (n = 213)	p-value
Female sex, n (%)	93 (43.7)	93 (43.7)	1.000
Age (years)	36.7 ± 7.6	36.2 ± 6.6	0.452
Current smoking, n (%)	126 (59.2)	95 (44.6)	χ²=10.3, **p=0.001**
Physical activity, yes, n (%)	63 (29.6)	69 (32.4)	χ²=0.3, p=0.606
BMI (kg/m²)	30.3 ± 5.7	28.7 ± 7.3	0.410
WC (cm)	108.8 ± 18.9	97.4 ± 20.5	**<0.001**
CRP (mg/L)	4.1 ± 4.5	1.2 ± 0.9	**<0.001**
FLI (score)	69.3 ± 28.5	55.7 ± 32.5	**<0.001**
VAI (score)	3.4 ± 2.6	2.3 ± 1.3	**<0.001**

BMI, Body Mass Index; WC, Waist Circumference; CRP, C-Reactive Protein; FLI, Fatty Liver Index; VAI, Visceral Adiposity Index.

A bold p-value indicates statistical significance.

By design, sex and age did not differ between groups. Compared with controls, patients with psoriasis showed a higher prevalence of current smoking (59.2% *vs* 44.6%; p = 0.001). No significant differences in physical activity were observed (p = 0.606).

Patients with psoriasis had significantly higher WC (108.8 ± 18.9 *vs* 97.4 ± 20.5 cm; p < 0.001), CRP levels (4.1 ± 4.5 *vs* 1.2 ± 0.9 mg/L; p < 0.001), FLI score (69.3 ± 28.5 *vs* 55.7 ± 32.5 score; p < 0.001), and VAI score (3.4 ± 2.6 *vs* 2.3 ± 1.3 score; p < 0.001). BMI did not differ significantly between groups (p = 0.410).

Patients with psoriasis had PASI scores ranging from 0.20 to 28.0; accordingly, 143 (67.1%) had mild disease, 56 (26.3%) moderate disease, and 14 (6.6%) severe psoriasis.

Sleep characteristics and chronotype of patients with psoriasis and controls are reported in **Table 2**.

**Table 2:** Sleep quality and chronotype in patients with psoriasis and matched healthy controls.

Parameter	Psoriasis (n = 213)	Controls (n = 213)	p-value
PSQI (score)	9.3 ± 4.9	6.8 ± 4.7	**<0.001**
Good sleepers, n (%)	53 (24.9)	104 (48.8)	χ²=24.3, **p<0.001**
Poor sleepers, n (%)	160 (75.1)	109 (51.2)	
MEQ (score)	39.2 ± 19.8	43.1 ± 19.2	**0.032**
Morning chronotype, n (%)	48 (22.5)	62 (29.1)	χ²=3.4, p=0.187
Intermediate chronotype, n (%)	18 (8.5)	22 (10.3)	
Evening chronotype, n (%)	147 (69.0)	129 (60.6)	

PSQI, Pittsburgh Sleep Quality Index; MEQ, Morningness–Eveningness Questionnaire.

A bold p-value indicates statistical significance.

PSQI scores were significantly higher in patients with psoriasis compared with controls (9.3 ± 4.9 *vs* 6.8 ± 4.7 score; p < 0.001), reflecting poorer sleep quality. Accordingly, the proportion of good sleepers was significantly lower in patients with psoriasis (24.9% *vs* 48.8%; p < 0.001), while poor sleepers were more prevalent (75.1% *vs* 51.2%).

Patients with psoriasis had a lower MEQ score than controls (39.2 ± 19.8 *vs* 43.1 ± 19.2 score; p = 0.032), indicating a tendency toward an evening chronotype. However, the overall distribution of chronotype categories (morning, intermediate, and evening) did not differ significantly between patients and controls (p = 0.187).

Baseline characteristics of patients with psoriasis and controls stratified by sleep quality (PSQI score ≤ 5 *vs* > 5) are shown in **Table 3**.

**Table 3:** Characteristics of patients with psoriasis and matched healthy controls according to sleep quality.

Parameter	Psoriasis		p-value
	Good sleepers (PSQI ≤ 5) N=53, 24.9%	Poor sleepers (PSQI > 5) N=160, 75.1%	
Female sex, n (%)	15 (28.3)	78 (48.8)	χ²=6.8, **p=0.009**
Age (years)	38.0 ± 6.3	36.3 ± 7.9	0.150
Current smoking, n (%)	9 (17.0)	117 (73.1)	χ²=51.9, **p<0.001**
Physical activity, yes, n (%)	32 (60.4)	31 (19.4)	χ²=32.1, **p<0.001**
BMI (kg/m²)	28.5 ± 4.8	30.8 ± 5.8	**0.008**
WC (cm)	99.4 ± 17.0	111.9 ± 18.5	**<0.001**
CRP (mg/L)	3.3 ± 4.1	4.4 ± 4.6	0.140
FLI (score)	55.7 ± 28.2	73.8 ± 27.2	**<0.001**
VAI (score)	2.2 ± 1.3	3.8 ± 2.8	**<0.001**
Controls			
	Good sleepers (PSQI ≤ 5) N=104, 48.8%	Poor sleepers (PSQI > 5) N=109, 51.2%	p-value
Female sex, n (%)	46 (44.2)	47 (43.1)	χ²=0.03, p=0.870
Age (years)	34.3 ± 6.2	38.0 ± 6.5	**<0.001**
Current smoking, n (%)	30 (28.8)	65 (59.6)	χ²=20.4, **p<0.001**
Physical activity, yes, n (%)	46 (44.2)	23 (21.1)	χ²=13.0, **p<0.001**
BMI (kg/m²)	24.3 ± 3.1	34.9 ± 6.3	**<0.001**
WC (cm)	84.0 ± 13.0	110.1 ± 18.0	**<0.001**
CRP (mg/L)	1.1 ± 1.0	1.4 ± 0.9	**0.035**
FLI (score)	31.1 ± 21.8	79.1 ± 21.9	**<0.001**
VAI (score)	1.8 ± 0.8	2.8 ± 1.4	**<0.001**

BMI, Body Mass Index; WC, Waist Circumference; CRP, C-Reactive Protein; FLI, Fatty Liver Index; VAI, Visceral Adiposity Index.

A bold p-value indicates statistical significance.

Among patients with psoriasis, poor sleepers were more frequently female (48.8% *vs* 28.3%; p = 0.009) and current smokers (73.1% *vs* 17.0%; p < 0.001), less physically active (19.4% *vs* 60.4%; p < 0.001), and showed higher BMI (30.8 ± 5.8 *vs* 28.5 ± 4.8 kg/m²; p = 0.008), WC (111.9 ± 18.5 *vs* 99.4 ± 17.0 cm; p < 0.001), FLI score (73.8 ± 27.2 *vs* 55.7 ± 28.2 score; p < 0.001), and VAI score (3.8 ± 2.8 *vs* 2.2 ± 1.3 score; p < 0.001). Physical activity was also higher among good sleepers (60.4% *vs* 19.4%; p < 0.001).

Among controls, poor sleepers were older (38.0 ± 6.5 *vs* 34.3 ± 6.2 years; p < 0.001), more frequently current smokers (59.6% *vs* 28.8%; p < 0.001), less physically active (21.1% *vs* 44.2%; p < 0.001), and had higher BMI (34.9 ± 6.3 *vs* 24.3 ± 3.1 kg/m²; p < 0.001), WC (110.1 ± 18.0 *vs* 84.0 ± 13.0 cm; p < 0.001), FLI score (79.1 ± 21.9 *vs* 31.1 ± 21.8 score; p < 0.001), VAI score (2.8 ± 1.4 *vs* 1.8 ± 0.8 score; p < 0.001), and CRP levels (1.4 ± 0.9 *vs* 1.1 ± 1.0 mg/L; p = 0.035).

Characteristics of patients with psoriasis and controls stratified by chronotype are shown in **Table 4**.

**Table 4:** Characteristics of patients with psoriasis and matched healthy controls according to chronotype category.

	Psoriasis			p-value
	Morning chronotype N=48, 22.5%	Intermediate chronotype N=18, 8.5%	Evening chronotype N=147, 69.0%	
Female sex, n (%)	16 (33.3)	5 (27.8)	72 (49.0)	χ²=5.6, p=0.060
Age (years)	38.1 ± 7.1	33.5 ± 6.3	36.7 ± 7.8	0.088
Current smoking, n (%)	0	8 (44.4)	118 (90.3)	χ²=97.8, **p<0.001**
Physical activity, yes, n (%)	33 (68.8)	7 (38.9)	23 (15.6)	χ²=50.0, **p<0.001**
BMI (kg/m²)	27.5 ± 4.6	28.5 ± 3.5	31.4 ± 5.9 ^a^	**<0.001**
WC (cm)	96.7 ± 17.5	107.3 ± 14.5	113.3 ± 17.9 ^a^	**<0.001**
CRP (mg/L)	2.9 ± 3.9	2.1 ± 2.5	4.8 ± 4.7 a^,b^	**<0.001**
FLI (score)	48.2 ± 29.3	64.8 ± 25.9	76.8 ± 24.9 ^a^	**<0.001**
VAI (score)	2.0 ± 1.2	2.6 ± 1.6	4.0 ± 2.8 ^a^	**<0.001**
Controls				
	Morning chronotype N=62, 29.1%	Intermediate chronotype N=22, 10.3%	Evening chronotype N=129, 60.6%	p-value
Female sex, n (%)	28 (45.2)	14 (63.6)	51 (39.5)	χ²=4.53, p=0.104
Age (years)	35.8 ± 8.3	36.9 ± 7.0	37.2 ± 7.3	0.514
Current smoking, n (%)	16 (25.8)	6 (27.3)	73 (56.6)	χ²=19.0, **p<0.001**
Physical activity, yes, n (%)	33 (53.2)	10 (45.5)	26 (20.2)	χ²=22.9, **p<0.001**
BMI (kg/m²)	29.9 ± 5.2	30.5 ± 4.8	30.4 ± 6.1	0.845
**WC (cm)**	108.4 ± 19.5	111.2 ± 17.1	108.6 ± 19.0	0.819
CRP (mg/L)	1.4 ± 1.0 ^c^	0.8 ± 1.0	1.2 ± 0.9 ^b^	**0.002**
FLI (score)	18.6 ± 12.6 ^c^	30.9 ± 14.7	77.7 ± 19.2 a^,b^	**<0.001**
VAI (score)	1.7 ± 0.7	2.0 ± 1.1	2.6 ± 1.4 ^a^	**<0.001**

BMI, Body Mass Index; WC, Waist Circumference; CRP, C-Reactive Protein; FLI, Fatty Liver Index; VAI, Visceral Adiposity Index.

a evening versus morning chronotype (Bonferroni *post hoc*).

b evening versus intermediate chronotype (Bonferroni *post hoc*).

c morning versus intermediate chronotype (Bonferroni *post hoc*).

A bold p-value indicates statistical significance.

Among patients with psoriasis, evening chronotype was associated with a higher prevalence of current smoking (90.3%; p < 0.001) and lower physical activity (15.6%; p < 0.001) compared with morning and intermediate chronotypes. Evening chronotype was also associated with higher BMI (31.4 ± 5.9 kg/m²), WC (113.3 ± 17.9 cm), CRP levels (4.8 ± 4.7 mg/L), FLI score (76.8 ± 24.9 score), and VAI score (4.0 ± 2.8 score) compared with morning chronotype (p < 0.001, Bonferroni *post-hoc*) and with intermediate chronotype for CRP levels, FLI score, and VAI score (p < 0.001, Bonferroni *post-hoc*).

Among controls, evening chronotype was similarly associated with a higher prevalence of current smoking (56.6%; p < 0.001) and lower physical activity (20.2%; p < 0.001) compared with morning and intermediate chronotypes. *Post-hoc* comparisons indicated significantly higher CRP levels (evening *vs* morning, p = 0.002), FLI score (evening *vs* morning and evening *vs* intermediate, p < 0.001), and VAI score (evening *vs* morning, p < 0.001) in evening chronotypes.

**Figure 2:**
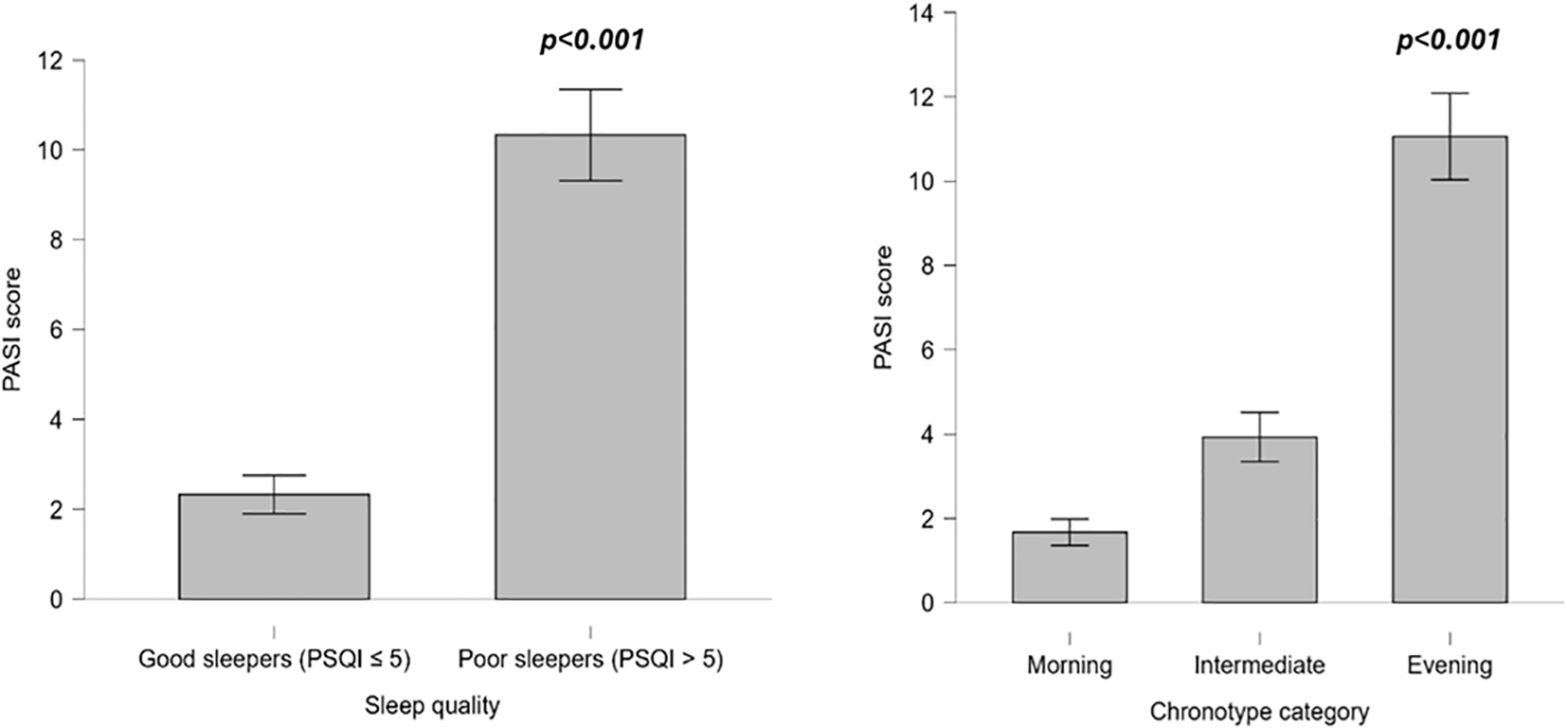
PASI score according to sleep quality and chronotype category in patients with psoriasis. PASI, Psoriasis Area and Severity Index; PSQI, Pittsburgh Sleep Quality Index.

**Figure 2** shows PASI scores in patients with psoriasis according to sleep quality and chronotype.

Poor sleepers (PSQI score > 5) had significantly higher PASI scores than good sleepers (PSQI score ≤ 5; 10.3 ± 6.5 *vs* 2.3 ± 1.6 score; p < 0.001).

When stratified by chronotype, evening chronotypes had the highest PASI score (11.1 ± 6.3 score), significantly higher than both morning (1.7 ± 1.1 score) and intermediate chronotypes (3.9 ± 1.2 score; both p < 0.001, Bonferroni *post-hoc*). No significant difference was observed between morning and intermediate chronotypes (p = 0.369).

Correlation analysis between PASI score and study parameters are shown in **Table 5**.

**Table 5:** Correlation analysis between PASI score and study parameters.

Parameter	PASI score	
	Pearson’s r	p-value
Age (years)	−0.041	0.547
BMI (kg/m²)	0.383	**<0.001**
WC (cm)	0.478	**<0.001**
PSQI (score)	0.766	**<0.001**
MEQ (score)	−0.781	**<0.001**
CRP (mg/L)	0.435	**<0.001**
FLI (score)	0.457	**<0.001**
VAI (score)	0.627	**<0.001**

PASI, Psoriasis Area and Severity Index; BMI, Body Mass Index; WC, Waist Circumference; PSQI, Pittsburgh Sleep Quality Index; MEQ, Morningness–Eveningness Questionnaire; CRP, C-Reactive Protein; FLI, Fatty Liver Index; VAI, Visceral Adiposity Index.

A bold p-value indicates statistical significance.

PASI score was positively correlated with BMI (r = 0.383, p < 0.001), WC (r = 0.478, p < 0.001), PSQI score (r = 0.766, p < 0.001), CRP levels (r = 0.435, p < 0.001), FLI score (r = 0.457, p < 0.001), and VAI score (r = 0.627, p < 0.001), and inversely correlated with MEQ score (r = −0.781, p < 0.001).

A stepwise multiple linear regression was performed with PASI score as the dependent variable (**Table 6**).

**Table 6:** Multivariable linear regression analysis identifying independent predictors of psoriasis severity (PASI score).

Dependent variable: PASI score					
Predictor	B	SE	β	t	p-value
Constant	−1.359	2.315	–	−0.587	0.558
MEQ (score)	−0.107	0.018	−0.316	−5.826	**<0.001**
PSQI (score)	0.445	0.057	0.330	7.871	**<0.001**
FLI (score)	−0.037	0.016	−0.158	−2.355	**0.019**
VAI (score)	0.599	0.100	0.233	6.017	**<0.001**
CRP (mg/L)	0.183	0.049	0.124	3.750	**<0.001**
Age (years)	0.019	0.027	0.021	0.689	0.492
BMI (kg/m²)	0.099	0.070	0.085	1.418	0.158
WC (cm)	0.045	0.023	0.129	1.980	**0.049**
Current smoking (yes/no)	1.637	0.625	0.121	2.619	**0.009**
Physical activity (yes/no)	−0.420	0.544	−0.029	−0.772	0.441
Model fit: R = 0.903; R² = 0.815; Adjusted R² = 0.806; SEE = 2.93					

PASI, Psoriasis Area and Severity Index; SE, Standard Error; MEQ, Morningness–Eveningness Questionnaire; PSQI, Pittsburgh Sleep Quality Index; FLI, Fatty Liver Index; VAI, Visceral Adiposity Index; CRP, C-Reactive Protein; BMI, Body Mass Index; WC, Waist Circumference; SEE, Standard Error of the Estimate.

A bold p-value indicates statistical significance.

Candidate predictors included age, BMI, WC, MEQ score, PSQI score, CRP levels, FLI score, VAI score, smoking status, and physical activity. In the fully adjusted multivariable model, MEQ score, PSQI score, FLI score, VAI score, CRP levels, WC, and smoking status emerged as independent predictors of PASI score, whereas age, BMI, and physical activity were not significantly associated.

The model explained approximately 81% of the variance in PASI score (adjusted R² = 0.806). MEQ score showed the strongest independent inverse association with PASI score (p < 0.001). PSQI score (p < 0.001), VAI score (p < 0.001), CRP levels (p < 0.001), WC (p = 0.049), and smoking status (p = 0.009) were positively associated with PASI score, while FLI score showed a modest inverse association (p = 0.019).

**Figure 3:**
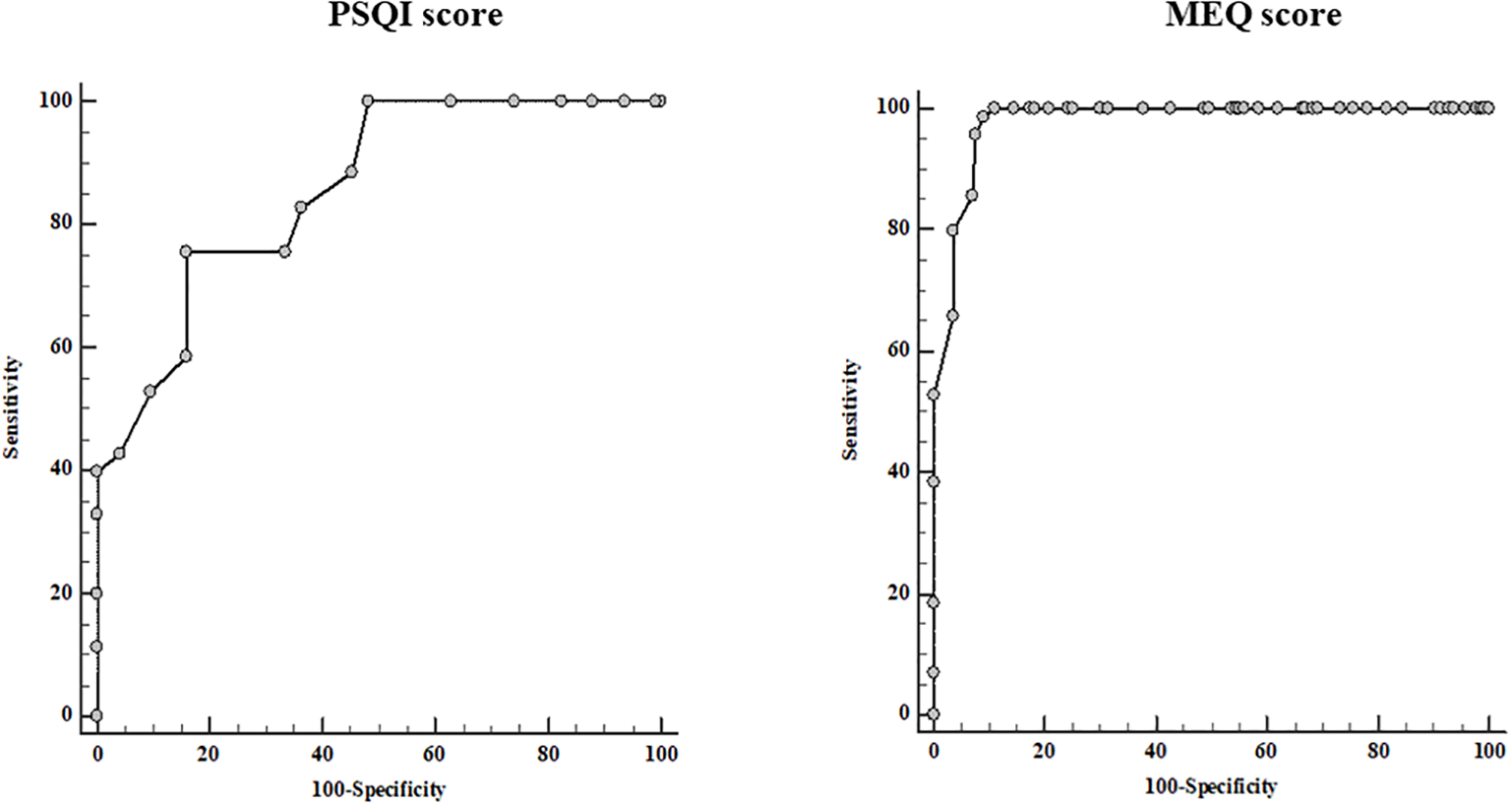
Receiver operator characteristic (ROC) for the cut-off value PSQI and MEQ scores predictive of a moderate-to-severe psoriasis clinical severity. PASI, Psoriasis Area and Severity Index; PSQI, Pittsburgh Sleep Quality Index; MEQ, Morningness–Eveningness Questionnaire.

ROC curve analysis (**Figure 3**) showed that a PSQI score > 10 predicted a moderate-to-severe PASI score with 75.7% sensitivity and 83.9% specificity (AUC 0.856, 95% CI 0.801–0.900, p < 0.001), while a MEQ score ≤ 25 predicted a moderate-to-severe PASI score with 98.6% sensitivity and 90.9% specificity (AUC 0.979, 95% CI 0.949–0.993, p < 0.001).

## 4 Discussion

In this cross-sectional case-control study, we investigated the relationships among psoriasis clinical severity, sleep quality, chronotype, and cardiometabolic indices in a well-characterized cohort of patients with mild-to-severe psoriasis and healthy controls.

In our cohort, despite comparable BMI between groups, patients with psoriasis exhibited significantly higher waist circumference, CRP levels, FLI, and VAI scores, suggesting greater visceral adiposity and systemic inflammation compared to controls. This finding supports the notion that psoriasis is not only a skin disease but also a systemic inflammatory condition, often accompanied by metabolic dysregulation (20). The positive correlations of PASI score with WC, CRP levels, FLI, and VAI scores reinforce the link between visceral adiposity, hepatic steatosis, and disease clinical severity.

Patients with psoriasis also had significantly poorer sleep quality, as reflected by higher PSQI scores, and a tendency toward evening chronotype, with lower MEQ scores compared with controls. Correspondingly, the proportion of poor sleepers and evening chronotypes was more prevalent among patients with psoriasis. These findings suggest that sleep disturbances and an evening circadian preference are common in psoriasis and should be assessed alongside traditional metabolic risk factors. Several mechanisms may underlie these observations. Chronic systemic inflammation, characteristic of psoriasis, can disrupt circadian regulation and alter melatonin and cortisol rhythms, contributing to sleep disturbances and a preference for evening activity (21). Nocturnal symptoms such as pruritus and pain may further impair sleep (22), while metabolic dysregulation, including increased visceral adiposity, may exacerbate circadian misalignment (11). Behavioral factors, including higher prevalence of smoking and lower physical activity among evening chronotypes, may reinforce these patterns (23). Additionally, psychological stress and altered light exposure can modulate sleep–wake cycles, creating a bidirectional relationship whereby disrupted sleep may also worsen psoriatic inflammation, perpetuating a cycle of poor sleep and increased disease severity (11).

Focusing on patients with psoriasis, poor sleepers were more frequently female and current smokers and exhibited higher BMI, WC, FLI score, and VAI score compared with good sleepers, indicating that sleep disruption clusters with adverse metabolic and inflammatory profiles. Similarly, patients with an evening chronotype exhibited higher BMI, WC, CRP levels, FLI score, and VAI score compared to those with morning or intermediate chronotypes, indicating clustering of adverse metabolic and inflammatory profiles. Evening chronotype was also associated with higher prevalence of smoking and lower physical activity. These findings are in line with previous evidence showing that sleep disturbances and chronodisruption are linked to obesity, visceral adiposity, insulin resistance, dyslipidemia, and elevated inflammatory markers (12, 24, 25). Mechanistically, sleep disruption and evening chronotype can alter hypothalamic–pituitary–adrenal (HPA) axis activity (26, 27) and increase and promote pro-inflammatory cytokine release (e.g., interleukin-6, tumor necrosis factor-α, and CRP levels) (28, 29). Chronodisruption can also impair glucose and lipid metabolism, disrupt appetite regulation, and favor adiposity accumulation, collectively contributing to an adverse metabolic-inflammatory profile (30).

These sleep and circadian factors were associated with differences in clinical severity. Poor sleepers (PSQI score > 5) exhibited significantly higher PASI scores than good sleepers (PSQI score ≤ 5), while evening chronotypes had the highest PASI score, significantly exceeding both morning and intermediate chronotypes. These observations are consistent with evidence that sleep disruption and circadian misalignment are linked to systemic inflammation and immune dysregulation, key drivers of psoriasis pathophysiology (12, 29). In addition, in an experimental model of psoriasis, it has been suggested that sleep disturbance may contribute to the exacerbation of psoriasis via the dysregulation of the immune mechanisms in the epidermal barrier (31). Moreover, evening chronotypes often exhibit behavioral patterns—such as later meal timing, reduced physical activity, and higher rates of smoking—that may be associated with greater metabolic and inflammatory stress, thereby potentially contributing to greater disease severity (12).

A major finding of our study is that sleep quality and chronotype were the strongest independent variables associated with PASI score, even compared with other factors retained in the stepwise regression model, including sleep quality (PSQI score), visceral adiposity (VAI score), systemic inflammation (CRP levels), WC, and smoking. In particular, it is plausible that chronotype reflects an underlying circadian predisposition linked to physiological systems relevant to psoriasis, such as immune function, hormonal rhythms, and inflammatory cytokine secretion (7). Chronotype can be modulated by environmental and lifestyle factors but also represents a relatively stable, individual-level circadian tendency, which may exert cumulative effects on metabolic and inflammatory pathways (7). In contrast, PSQI score, VAI score, CRP levels, and WC capture specific but potentially more transient aspects of sleep quality, adiposity, and systemic inflammation, which may partly explain why their independent predictive value for PASI score was lower than that of chronotype in our cohort.

ROC curve analysis further supported the clinical relevance of these parameters. A PSQI score > 10 and a MEQ score ≤ 25 predicted moderate-to-severe PASI, confirming the potential utility of sleep and circadian metrics for clinical risk stratification. Importantly, both questionnaires are simple, non-invasive, and easy to implement in routine clinical practice (16, 17), making them practical tools for identifying patients at higher risk of severe psoriasis.

## 5 Strengths and limitations

This study has several important strengths. First, it is among the first to comprehensively examine the interrelationships between psoriasis clinical severity, sleep quality, chronotype, visceral adiposity, and systemic inflammation within a single, well-characterized cohort. The integrated assessment of sleep–circadian parameters (PSQI and MEQ scores), validated surrogate indices of visceral adiposity and hepatic steatosis (VAI and FLI scores), and inflammatory markers provides a robust and clinically meaningful framework for understanding psoriasis as a systemic disorder extending beyond its cutaneous manifestations.

Second, psoriasis clinical severity was assessed using the PASI score by a qualified dermatologist, ensuring high clinical accuracy and reducing inter-observer variability. Importantly, sleep quality and chronotype questionnaires were administered by trained and qualified nutritionists rather than being self-completed by patients, minimizing reporting bias and enhancing the reliability and consistency of the collected data. This methodological approach represents a notable strength compared with many previous studies relying solely on self-reported assessments.

Third, all participants were recruited from the same geographical area (Naples, Italy), thereby sharing similar cultural, dietary, and lifestyle habits. This geographical homogeneity reduces potential confounding related to dietary patterns and environmental factors and strengthens the internal validity of the observed associations between sleep–circadian characteristics, cardiometabolic indices, and psoriasis clinical severity.

Furthermore, the inclusion of matched controls and the careful phenotyping of patients across a wide spectrum of disease clinical severity enhance the robustness of the findings. Notably, the demonstration of increased visceral adiposity and inflammatory burden in patients with psoriasis despite comparable BMI underscores the importance of using WC and adiposity indices beyond BMI alone.

The application of multivariable regression models and ROC curve analysis allowed the identification of sleep quality and chronotype as independent and clinically relevant predictors of PASI score, beyond traditional metabolic and inflammatory risk factors. The finding that chronotype emerged as the strongest independent predictor of psoriasis clinical severity is novel and carries significant translational implications. Moreover, the use of simple, non-invasive, and validated questionnaires supports the feasibility of integrating sleep and chronotype assessment into routine outpatient clinical practice.

Overall, the strengths of this study—particularly its rigorous clinical assessment, standardized data collection, geographical homogeneity, and integrated analytical approach—provide a solid foundation for the interpretation of the findings and support the need for future longitudinal and interventional studies in this emerging area.

Our study also has several limitations. First, its cross-sectional design precludes causal inference, and therefore the directionality of the associations between sleep–circadian factors and psoriasis clinical severity cannot be established. Furthermore, although ROC analysis identified specific PSQI and MEQ thresholds associated with moderate-to-severe psoriasis, these findings were derived from the same sample used to build the models and were not externally validated. As such, the reported cut-offs and their diagnostic performance may reflect cohort-specific characteristics and should be confirmed in independent populations before clinical implementation. Second, although validated questionnaires were used to assess sleep quality and chronotype, these assessments, while administered by trained nutritionists, were self-reported and may be subject to recall or reporting bias. Direct objective measures of sleep and circadian rhythms, such as actigraphy, polysomnography, or hormonal profiling, were not performed, which may limit the precision of the observed associations. Future studies incorporating objective sleep and circadian monitoring are warranted to confirm and extend our findings. Third, direct imaging-based assessment of visceral adiposity and hepatic steatosis (e.g., magnetic resonance imaging, computed tomography, or ultrasound) was not available; however, the FLI and VAI scores are widely validated surrogate markers and provide reliable estimates of metabolic risk (18, 19).

Additionally, the study population was predominantly characterized by excess body weight, with mean BMI values in the overweight/obesity range in both patients and controls, which, while enhancing relevance in metabolically high-risk populations, may limit the generalizability of the findings to lean individuals with psoriasis. Residual confounding from unmeasured lifestyle, environmental, or psychosocial factors cannot be entirely excluded, despite adjustment for major metabolic and inflammatory variables.

Moreover, the exclusion of patients with psoriatic arthritis and those receiving systemic therapies or phototherapy may limit the generalizability of our findings to these important clinical subgroups. While this approach was adopted to reduce treatment-related confounding and isolate disease-related associations, patients with joint involvement or more severe psoriasis often represent a substantial proportion of the psoriatic population. Future studies including these patients are needed to determine whether sleep quality and chronotype play a similar role in modulating disease clinical severity in the context of systemic inflammation and advanced disease.

Furthermore, psychiatric symptoms (e.g., depression, anxiety) and sleep disorders such as obstructive sleep apnea were not assessed, and their potential confounding or mediating effects on the relationships between sleep quality, chronotype, and psoriasis clinical severity cannot be excluded. Future studies should incorporate screening for mood disorders and sleep disorders to clarify the direct impact of sleep and circadian disruption on psoriasis outcomes.

Finally, while self-reported data were complemented by administration from trained nutritionists and PASI scoring by a qualified dermatologist, some measurement variability and bias inherent to questionnaire-based data collection cannot be completely ruled out.

## 5 Conclusions

In conclusion, this study provides the first evidence that sleep quality and chronotype are independently associated with the clinical psoriasis severity, irrespective of adiposity and traditional metabolic risk markers. Beyond their association with visceral adiposity and systemic inflammation, poor sleep quality and an evening chronotype emerged as clinical correlates of disease clinical severity, underscoring the relevance of circadian preference as a novel and clinically meaningful factor in psoriasis. These findings primarily apply to patients with psoriasis with increased adiposity and metabolic burden, and further studies are warranted to determine whether similar associations are present in lean individuals with psoriasis.

## Data Availability

The raw data supporting the conclusions of this article will be made available by the authors, without undue reservation.
